# Posterior intra-articular fixation stabilizes both primary and secondary sacroiliac joints: a cadaveric study and comparison to lateral trans-articular fixation literature

**DOI:** 10.1186/s13018-023-03886-3

**Published:** 2023-06-03

**Authors:** Dawood Sayed, Kasra Amirdelfan, Corey Hunter, Oluwatodimu Richard Raji

**Affiliations:** 1 The University of Kansas Medical Center, Kansas City, KS USA; 2 IPM Medical Group, Walnut Creek, CA USA; 3 Ainsworth Institute of Pain Management, New York, NY USA; 4 Medical Device Development, 2390 Mission Street, San Francisco, CA 94110 USA

**Keywords:** Range of motion, Arthrodesis, Allograft, Unilateral, Bilateral, Compression, Distraction, Interference, Interposition

## Abstract

**Background:**

Posterior and lateral techniques have been described as approaches to sacroiliac joint arthrodesis. The purpose of this study was to compare the stabilizing effects of a novel posterior stabilization implant and technique to a previously published lateral approach in a cadaveric multidirectional bending model. We hypothesized that both approaches would have an equivalent stabilizing effect in flexion–extension and that the posterior approach would exhibit better performance in lateral bending and axial rotation. We further hypothesized that unilateral and bilateral posterior fixation would stabilize both the primary and secondary joints.

**Methods:**

Ranges of motion (RoMs) of six cadaveric sacroiliac joints were evaluated by an optical tracking system, in a multidirectional flexibility pure moment model, between ± 7.5 N-m applied moment in flexion–extension, lateral bending, and axial rotation under intact, unilateral fixation, and bilateral fixation conditions.

**Results:**

Intact RoMs were equivalent between both samples. For the posterior intra-articular technique, unilateral fixation reduced the RoMs of both primary and secondary joints in all loading planes (flexion–extension RoM by 45%, lateral bending RoM by 47%, and axial RoM by 33%), and bilateral fixation maintained this stabilizing effect in both joints (flexion–extension at 48%, lateral bending at 53%, and axial rotation at 42%). For the lateral trans-articular technique, only bilateral fixation reduced mean RoM of both primary and secondary sacroiliac joints, and only under flexion–extension loads (60%).

**Conclusion:**

During flexion–extension, the posterior approach is equivalent to the lateral approach, while producing superior stabilization during lateral bend and axial rotation.

## Background

Lower back pain (LBP) has proven to be a burdensome health issue as it is a leading cause of disability worldwide [1]. Up to 38% of LBP incidence occurs as a result of sacroiliac (SI) joint degeneration or inflammation [2]. In such cases, pain is likely to be induced by joint motion and therefore treatment involves stabilizing and fusing the joint [2–5]. Methods for fixation of the sacroiliac joint were first described by Smith-Petersen for osteomyelitis, tuberculosis, and joint relaxation (hyperlaxity), with varying graft trajectories and later revised by Smith-Petersen and Rogers for traumatic and non-traumatic osteoarthritis [6, 7]. Anterior extra-articular, lateral trans-articular, and posterior intra-articular techniques have been implemented in the literature [2, 8–19]. Interest in the posterior sacroiliac fixation method has increased due to the increased distance of the implant placement from the neurovascular bundle that can be compromised as a result of anterior or caudal breach, or violation of the sacral neural foramen during fixation. However, this approach still bears the risk of damage to the cluneal nerves [17].

In the open anterior approach, the iliac muscle is retracted, and a plate and screws are used to fix the sacroiliac joint by coupling the sacrum to the iliac bone. However, this approach bears the risk of increasing detachment of the iliac muscle, lateral femoral cutaneous nerve injury, bleeding, and/or pelvic organ injuries, with previous investigators reporting 13.6–28.5% postoperative complication rates, and 53.8% incidence of postoperative contralateral sacroiliac joint pain [18, 19]. In the lateral approach, one or more implants are placed across the joint through an osseous iliac window, with the lateral portion of the implant in the ilium and the medial portion in the sacrum. This approach aims to immediately fix the joint while attempting to avoid disrupting the ligaments, although muscle damage remains a possible complication. The sacral nerves must be avoided, which results in a handful of possible regions for implant placement [8–13, 17]. Guide wires for screw fixation are normally placed at the superior, middle, and inferior regions of the joint [2]. The implants used in this approach are designed to promote fusion while coupling the medial and lateral portions of the joint, and/or compressing the joint [8–13]. In this approach, previous investigators have reported up to 88% fusion with 11–32.5% postoperative complication rates [20–22]. In the posterior approach, the joint is accessed posteriorly, distracted, and fixed as the implant is advanced anteriorly. Interposing the implant between the joint surfaces ensures that loads are transmitted through the implant, thus relieving pain due to stresses on joint cartilage and surrounding neural structures [23, 24]. The implants used in this approach, typically aim to distract and couple the medial and lateral portions of the joint [2, 14–17]. Previous investigators have also reported 31–89% fusion rates; however, no complications have been reported [20, 25]. These three approaches thus differ in their functional biomechanics for the fixation of the joint, which is a necessary component of the arthrodesis procedure.

The lateral approach remains the most common approach utilized for sacroiliac joint arthrodesis, as its efficacy in reducing joint motion has been immensely described in previous biomechanical studies [2, 8–13]. In contrast, there exist, currently, few biomechanical studies which assess the posterior approach [24].

## Methods

### Study aim and hypotheses

**Fig. 1:**
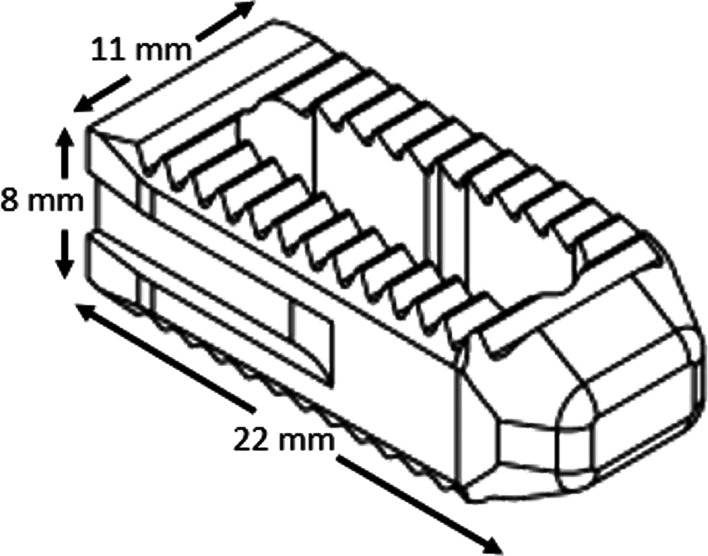
Illustration of the rectangular-shaped cortical allograft

**Fig. 2:**
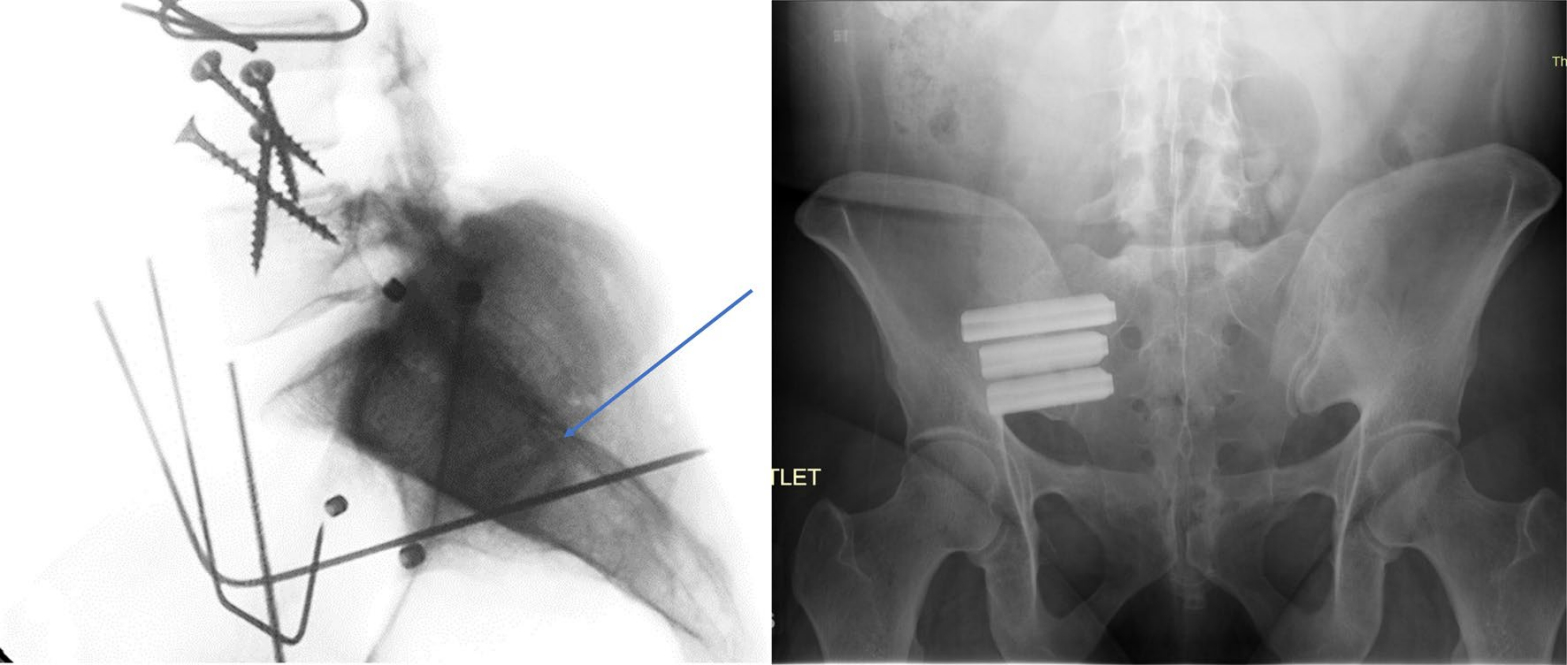
Post-implantation X-rays of posterior approach (left) and lateral approach (right) [26]

In this study, we aimed to assess and compare the motion reduction induced by the posterior intra-articular technique using a single novel interpositional cortical allograft implant (LinQ, PainTEQ, Fig. 1) in unilateral and bilateral fixation constructs to that reported for a lateral trans-articular technique (iFuse, SI-Bone) using three triangular rods, within the same biomechanical model and under the same applied loads [10]. We hypothesized that both techniques (shown in Fig. 2) would have an identical stabilizing effect during flexion–extension loads and that the posterior approach would generate greater reductions in motion during lateral bending, and axial rotation loads. We also hypothesized that both unilateral and bilateral fixations using the posterior technique would reduce the primary (ipsilateral) and secondary (contralateral) sacroiliac joints’ ranges of motion (RoMs).

### Specimen preparation

Six fresh-frozen cadaveric sacroiliac joints (four females and two males) from the L4 to pelvis were sourced through the American Association of Tissue Banks (AATB). A sample size of four sacroiliac joints was calculated using the following assumptions: 27% standard deviation, 95% significance, 80% power, and 50% effect size [9]. Each specimen was pre-screened for bone quality, and bone or sacroiliac joint disease using computerized tomographic (CT) and dual-energy x-ray absorptiometry (DEXA) scans, and any cadavers exhibiting joint fusion or osteoporotic bones were not sourced [27]. Each specimen was eviscerated and prepared by cleaning out soft tissue surrounding the pelvis. Care was taken to keep the sacroiliac ligaments and pubic symphysis intact.

Each ischium of the specimen was subsequently potted in fast-curing resin (Smooth-Cast 300Q, Smooth-On, Inc., Easton, Pennsylvania, USA) and aligned to fit the physiological pelvic orientation [28]. Alignment was done under fluoroscopy using Jamshidi bone biopsy needles and steel wires. The L4 vertebrae were potted after being rigidly affixed to the L5 using wood screws. The custom pure moment ring was attached to the potted L4 under fluoroscopy using the pubic symphysis to align the anterior posterior axis of the ring to the center of the vertebrae.

### Loading procedure

**Fig. 3:**
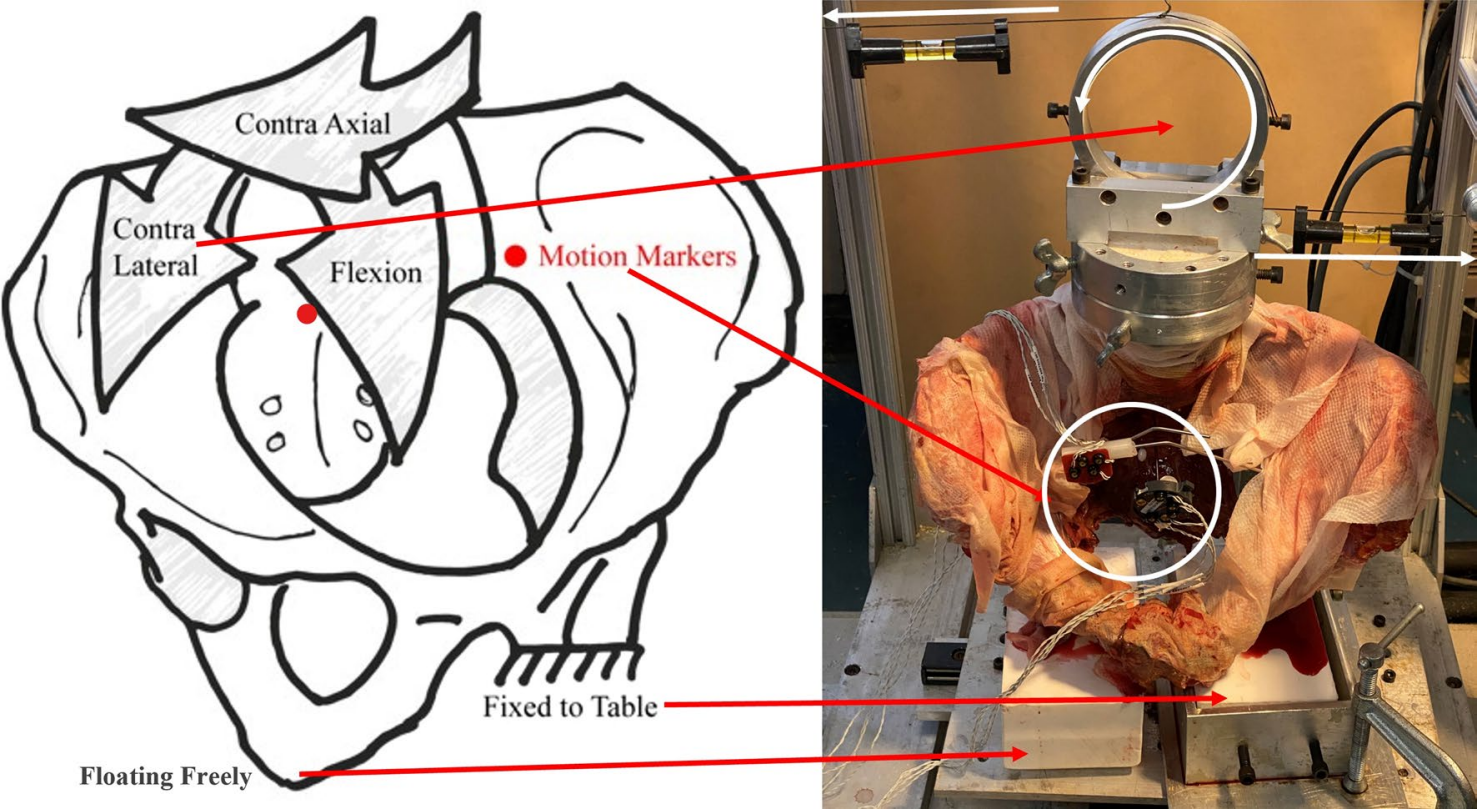
Illustration of the biomechanical model in the left single-leg stance. Pure moments were applied to the lumbar spine, as shown. Relative motions between the sacrum and the iliac were tracked with motion markers rigidly fixed to each bone. Not pictured are extension, ipsi axial rotation, and ipsi lateral bending arrows

The specimens were tested using a custom pure moment force ring that was connected, using a cable and pulley system, to a servo-hydraulic test frame (858 Mini Bionix II; MTS, Eden Prairie, MN, USA). The actuator applied tension on the cables which applied pure moment force on the specimen through the ring [29]. Each specimen was attached on a biomechanical testing fixture with the ischium of the tested joint fixed to the table, which was allowed to translate freely, to eliminate shear forces, and the other ischium free standing in order to simulate a single-leg stance [8–10]. The lumbar spine and sacrum were not constrained. Each specimen was loaded in three physiological planes: flexion/extension, left/right lateral bending, and left/right axial rotation (Fig. 3). The loading order of the specimens tested was randomly determined. Each specimen went through three preconditioning cycles where they were loaded from 0 N-m to 7.5 N-m, after which it was loaded in a fourth cycle and motion tracking data were recorded in 1.5 N-m intervals [8–11]. This was repeated for each of the six anatomical bending directions.

### Test order and joint fixation

**Fig. 4:**
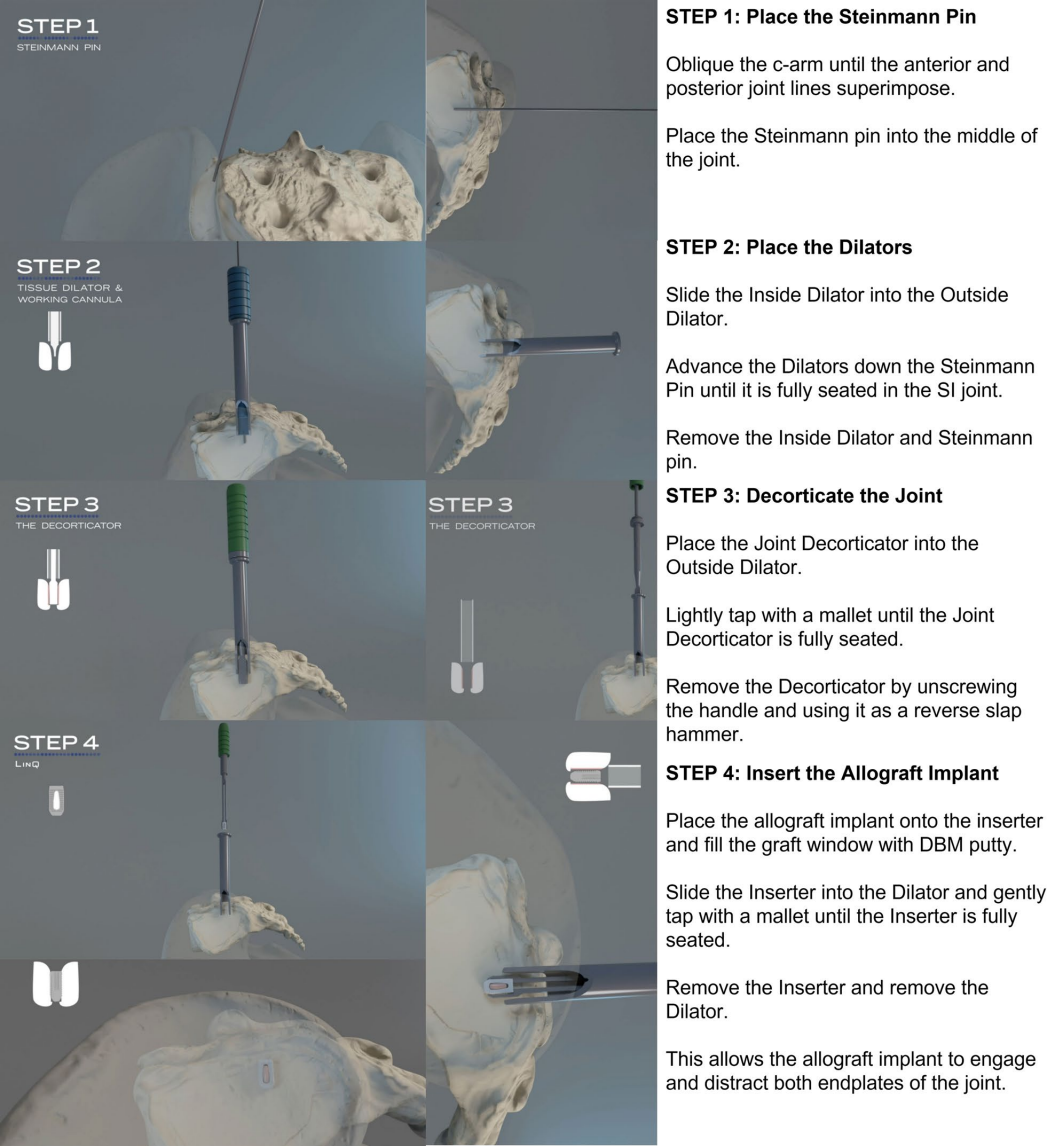
Surgical technique guide showing implantation technique of bone allograft implant

**Fig. 5:**
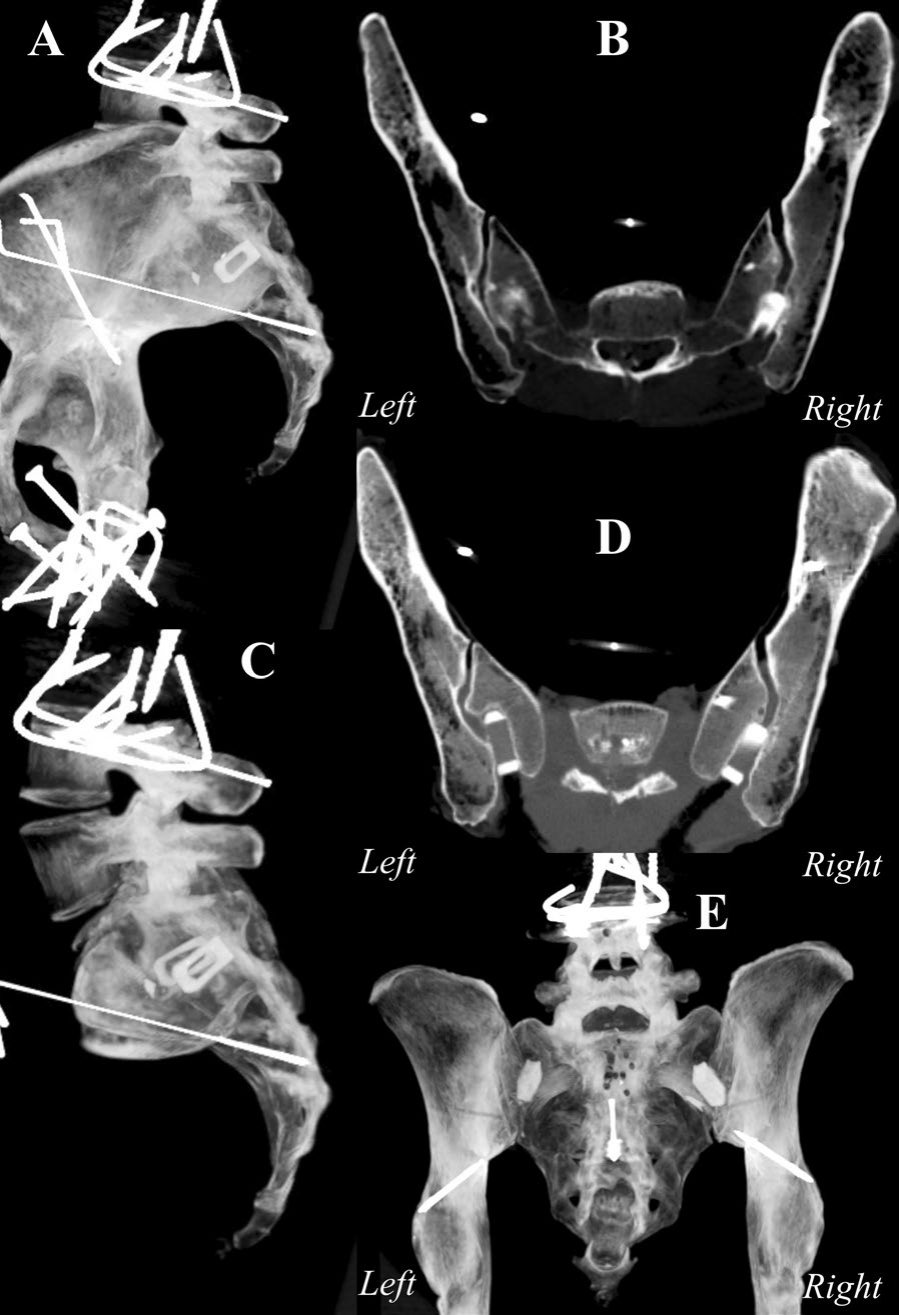
Maximum intensity projection (MIP) renderings, and images obtained from postoperative computed tomography (CT) scans upon fixation with bone allograft implant. Images are displayed are **A** sagittal pelvis [unilateral fixation], **B** axial pelvis [unilateral fixation], **C** sagittal sacrum only [bilateral fixation], **D** axial pelvis [bilateral fixation], **E** coronal pelvis [bilateral fixation]

Once a specimen was tested in the intact state, unilateral SI joint fixation was performed on the primary joint, and both the primary and secondary joints were tested, after which the bilateral SI joint fixation was performed by fixation of the contralateral joint, and both joints were retested. The primary joint was the joint first instrumented, and the secondary joint was the contralateral joint [10]. The primary joints (left/right) were chosen at random for each pelvis, and all fixations were performed according to the manufacturer’s instructions using a posterior approach (LinQ SI Joint Stabilization System; PainTEQ) (Fig. 4). The implants were placed between the S1 and S2 medial crests, at the level of the first sacral neural foramina, just below the PSIS and above the lateral sacral crest (Fig. 5).

### Motion analysis

To align the motion tracking system (3D Investigator and Optotrak systems by Northern Digital Inc., Waterloo, ON, Canada) to the physiological coordinate system of the specimen, a probe was used to digitize the extents of the sacral ala and the moment ring. The lateral axis was defined as the line connecting the left and right lateral extents of the moment ring (installed on the L4 vertebrae). The A–P axis was defined as the line connecting the anterior extent of the moment ring to the midpoint of lateral axis line. The combination of these two axes defined the transverse plane. And the origin was moved to the apex of the sacral ala. The Infrared motion markers were rigidly attached to the iliac brim and second sacral body [8–13].

Data from the markers were recorded during each loading interval at 100 Hz and a custom computer program executed by commercial software (MATLAB by MathWorks) extracted the peak motion observed during data collection from the raw data files. These data were used to find the range of motion in flexion–extension, lateral bending, and axial rotation in an intact, unilateral, and bilateral state for each specimen.

### Data analysis

The RoM of the intact state was compared to the predicate study using commercial software (JMP by SAS Institute in North Carolina, USA), to ensure no significant difference between both samples at *P* > 0.05, CI: 95% [10]. Data from the intact, unilateral, and bilateral constructs were compared using a paired t-test, to check for statistical significance at alpha equal to 0.05. Analysis is performed using repeated-measures ANOVA with post hoc comparisons using the Holm method. All data are reported as mean ± one standard deviation unless otherwise stated.

## Results

The age at the time of death ranged from 34 to 37 years, average body mass index was 26 ± 2, average lumbar t-score was 0.8 ± 0.6, and average lumbar bone density was 1.3 ± 0.2 g/cm^2^. Comparative analysis of the intact joints between the posterior and lateral samples (pooled, primary, and secondary) presented no significant difference when under the same loading conditions (Table 1). Likewise, no significant differences were observed in our samples, between primary and secondary.

**Table 1:** Results of comparative analysis of pooled data (left and right) for intact SI joints between posterior and lateral approach study samples [10]

Motion tested	Flexion–extension (Deg)		Lateral bending (Deg)		Axial rotation (Deg)	
Sample source	Posterior	Lateral	Posterior	Lateral	Posterior	Lateral
Primary joint	2.8 ± 1.5	2.8 ± 1.6	1.4 ± 0.6	1.1 ± 1.2	2.0 ± 0.7	1.8 ± 1.2
*p* value	0.997		0.660		0.784	
Secondary joint	3.0 ± 1.2	2.7 ± 1.9	1.5 ± 0.5	1.2 ± 1.2	2.7 ± 0.8	1.7 ± 1.4
*p* value	0.845		0.676		0.291	
Pooled joints	2.9 ± 1.2	2.8 ± 1.6	1.5 ± 0.5	1.2 ± 1.2	2.4 ± 0.8	1.8 ± 1.2
*p* value	0.880		0.517		0.517	

**Fig. 6:**
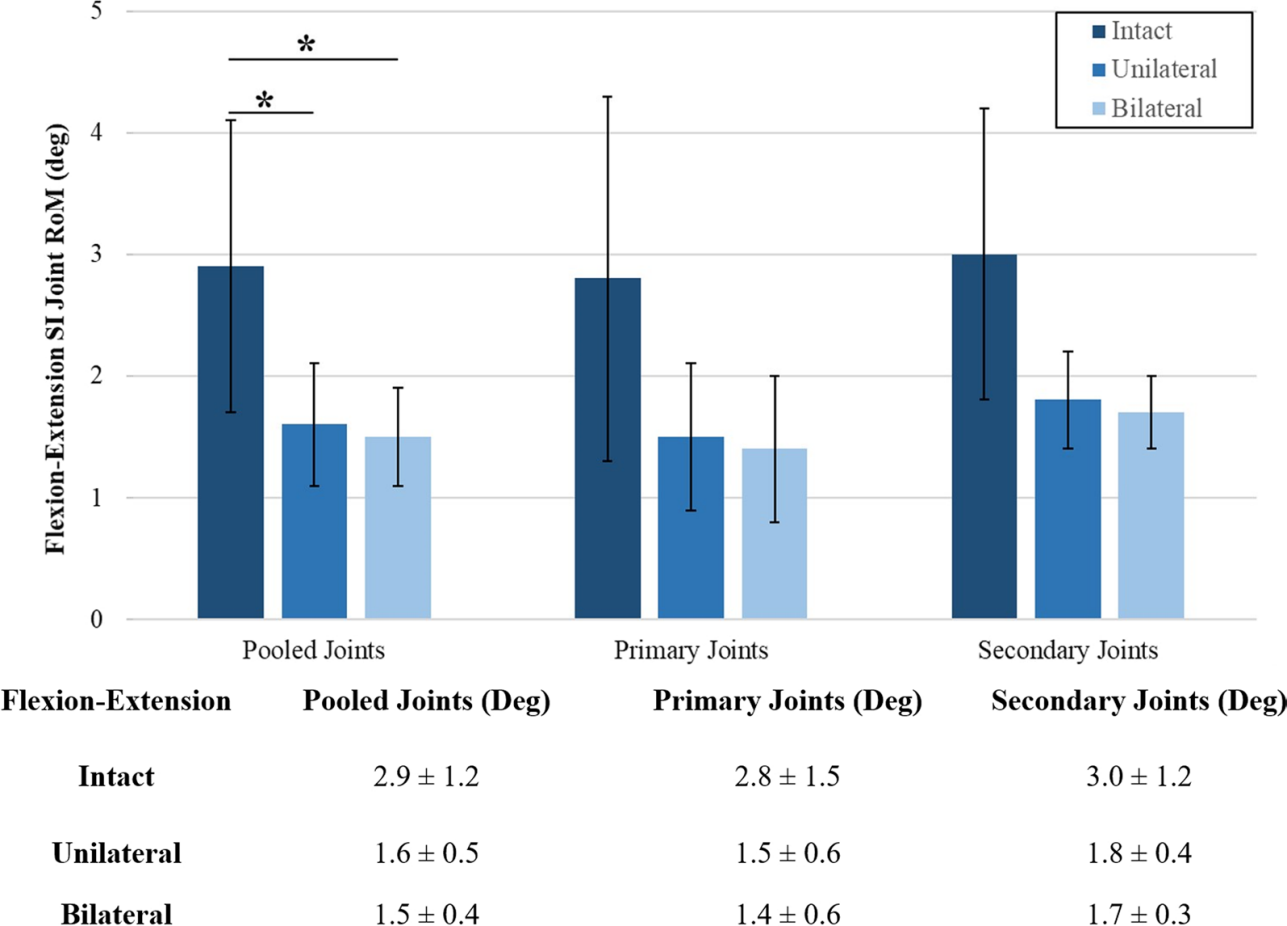
Ranges of rotational motion during flexion–extension. The y-axis lists the ranges of motion. The x-axis displays the joint group tested. The asterisk (*) indicates statistically significantly reduced motions at *p* < 0.05. The table shown lists the numbers used to create the chart. All data are represented as mean ± standard deviation

During flexion–extension (Fig. 6), unilateral fixation resulted in 34% ± 27% significant motion reduction in all pooled joints when compared to the intact condition (*p* = 0.049), bilateral fixation maintained this motion reduction in all joints at 39% ± 23% (*p* = 0.040), bilateral fixation reduced the range of motion slightly by 7% from unilateral fixation, but this reduction was not significant (*p* = 0.088).

**Fig. 7:**
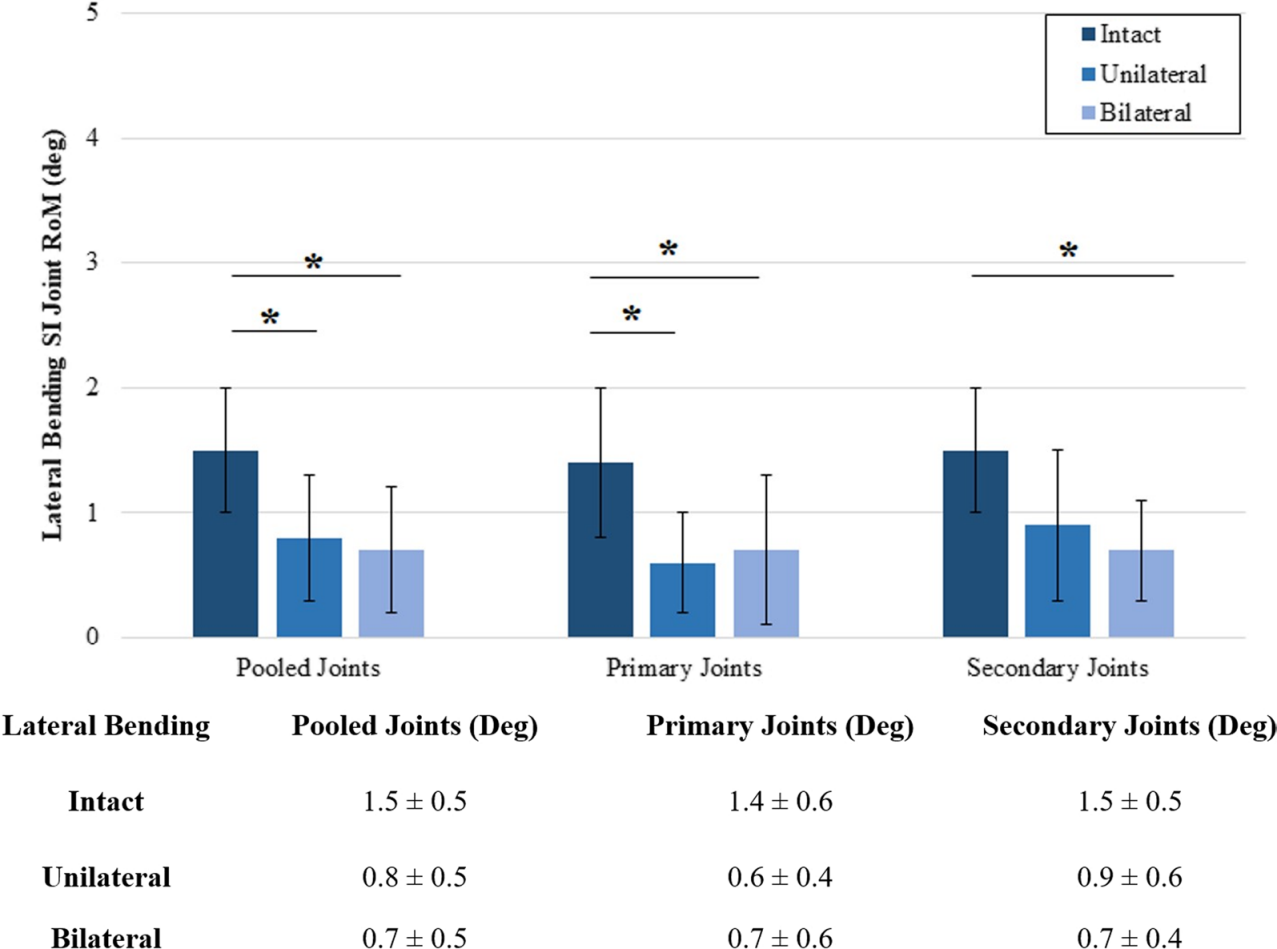
Ranges of rotational motion during lateral bending. The y-axis lists the ranges of motion. The x-axis displays the joint group tested. The asterisk (*) indicates statistically significantly reduced motions at *p* < 0.05. The table shown lists the numbers used to create the chart. All data are represented as mean ± standard deviation

During lateral bending (Fig. 7), unilateral fixation resulted in 51% ± 23% significant motion reduction in all pooled joints when compared to the intact condition (*p* = 0.004), and bilateral fixation maintained this motion reduction in all joints at 54% ± 23% (*p* = 0.001). Upon unilateral fixation, 62% ± 15% significant motion reduction was observed in the primary joint (*p* = 0.018) when compared to the intact motion. Upon bilateral fixation, 53% ± 21% significant motion reduction was maintained in the primary joints (*p* = 0.029), and 51% ± 11% significant motion reduction was observed in secondary joints (*p* = 0.046), when compared to the intact motion.

**Fig. 8:**
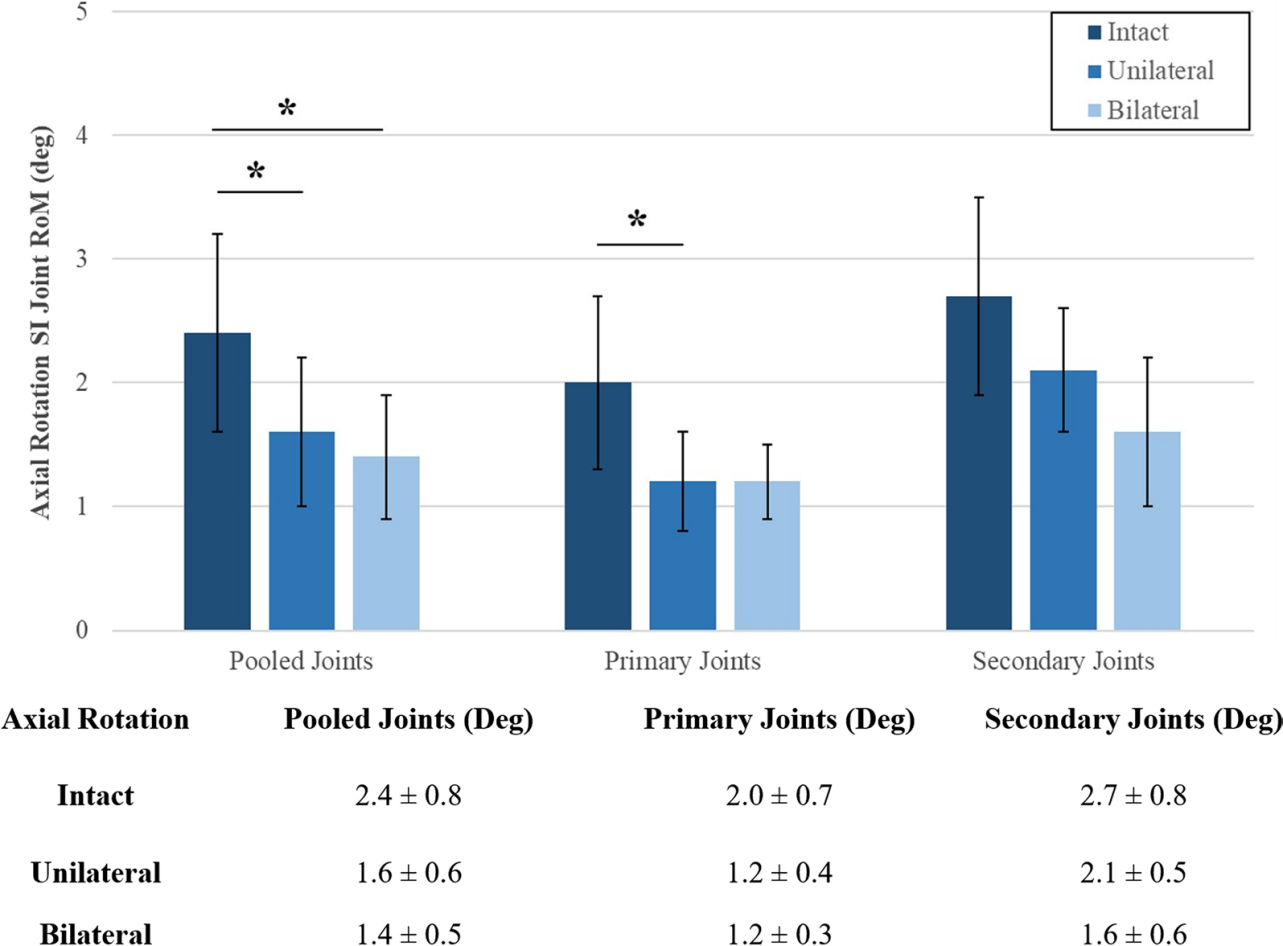
Ranges of rotational motion during axial rotation. The y-axis lists the ranges of motion. The x-axis displays the plane motion tested. The asterisk (*) indicates statistically significantly reduced motions at *p* < 0.05. The table shown lists the numbers used to create the chart. All data are represented as mean ± standard deviation

During axial rotation (Fig. 8), unilateral fixation resulted in 32% ± 14% significant motion reduction in all pooled joints when compared to the intact condition (*p* = 0.008) and bilateral fixation reduced this motion further in all joints to 39% ± 17% (*p* = 0.010). Upon unilateral fixation, 41% ± 2% significant motion reduction was observed in the primary joint (*p* = 0.043) when compared to the intact motion. Upon bilateral fixation, no significant difference was observed in the primary joints (*p* = 0.984), or in the secondary joint (*p* = 0.053) when compared to the unilateral fixation.

Bilateral fixation images (Fig. 5) indicated that there is left–right asymmetry in the joint surface/morphology. Even as both implants were placed at roughly the same height and trajectory, the implant was in line with the joint surface in the right joint which had a more consistent joint surface alignment in contrast to the left joint.

**Fig. 9:**
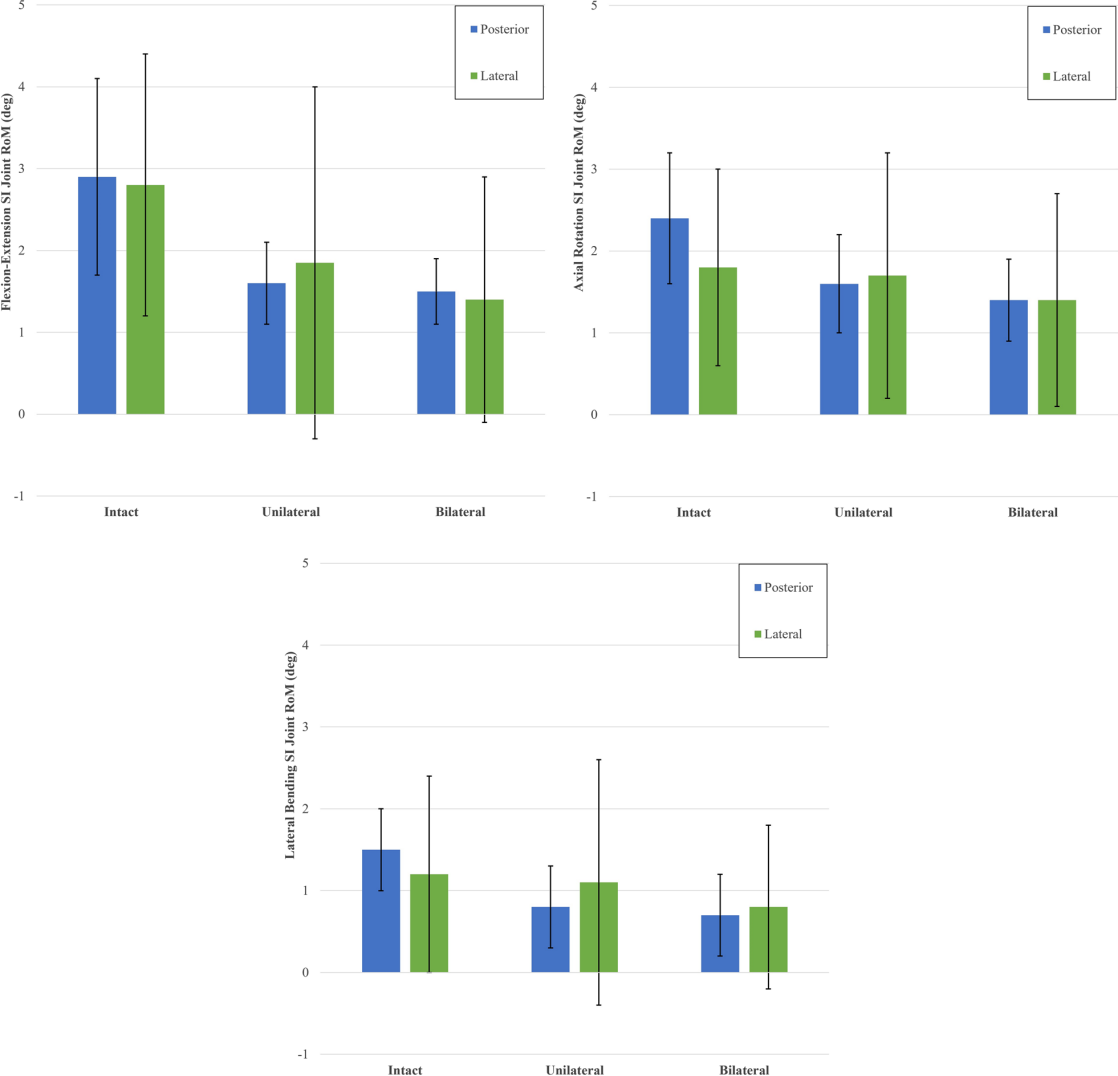
Comparison of posterior to lateral approach in unilateral and bilateral fixation constructs. The chart displays the pooled results of range of motion in both primary and secondary joints, during flexion/extension, axial rotation, and lateral bending. The y-axis lists the ranges of motion. The x-axis displays the plane motion tested

A comparison of pooled joints results between the posterior and lateral approaches is shown in Fig. 9 and Table 2. Comparing percent motion reduction from the intact to unilateral conditions, the posterior technique generated 32%, 450%, and 486% more motion reduction in comparison with the lateral technique in flexion–extension, axial rotation, and lateral bending, respectively. Comparing percentage motion reduction from the intact to bilateral conditions, the lateral technique generated 4% more motion reduction in flexion–extension compared to the posterior technique, while the posterior technique generated 91% and 61% more motion reduction in comparison with the lateral technique in axial rotation and lateral bending, respectively. The lateral technique reported 300%, 38%, and 108% more reduction between unilateral to bilateral conditions in flexion–extension, axial rotation, and lateral bending, respectively.

**Table 2:** Comparison of motion reduction in the posterior to lateral approach in unilateral and bilateral fixation constructs [10]

Test condition	Flexion–extension		Axial rotation		Lateral bending	
	Posterior	Lateral	Posterior	Lateral	Posterior	Lateral
Intact	2.9 ± 1.2	2.8 ± 1.6	2.4 ± 0.8	1.8 ± 1.2	1.5 ± 0.5	1.2 ± 1.2
Unilateral	1.6 ± 0.5	1.9 ± 2.2	1.6 ± 0.6	1.7 ± 1.5	0.8 ± 0.5	1.1 ± 1.5
Bilateral	1.5 ± 0.4	1.4 ± 1.5	1.4 ± 0.5	1.4 ± 1.3	0.7 ± 0.5	0.8 ± 1.0
% Reduction in mean intact versus unilateral	45%	34%	33%	6%	47%	8%
% Reduction in mean intact versus bilateral	48%	50%	42%	22%	53%	33%
% Reduction in mean unilateral versus bilateral	6%	24%	13%	18%	13%	27%

## Discussion

The goal of our study was to compare the effects of unilateral and bilateral fixations on sacroiliac joint mobility between a posterior and a lateral approach during single-leg stance in a cadaveric multidirectional bending flexibility model [10]. Our results indicate that posterior approach has a similar performance in stabilizing the SI joint during flexion–extension motions, and superior performance in stabilizing the SI joint during lateral bending and axial rotation motions, compared to published data using the lateral approach. We analyzed the intact motions of the joint between our samples and those of the previous research and found no significant differences in either primary, secondary, or pooled joint samples. Our intact results are in line with those of previous investigations which report the range from 4.5 ± 3.3 degrees to 2.3 ± 1.4 degrees for flexion–extension loading, 1.5 ± 1.5 degrees to 1.1 ± 0.8 degrees during left/right lateral bending, and 2.9 ± 2.1 degrees to 1.7 ± 0.8 degrees during left/right axial rotation [8, 9, 11–13].

The current and previous studies were performed using the same physiological model, preserving the pubic symphysis, to maintain an intact pelvic ring [10]. The posterior and lateral techniques produced similar motion reductions in flexion–extension, after unilateral and bilateral treatments. However, in axial rotation and lateral bending, the posterior approach generated 4.5 and 4.9 times more mean percent motion reductions upon unilateral treatment and subsequently 0.9 and 0.6 more mean percent motion reductions upon bilateral treatment.

**Fig. 10:**
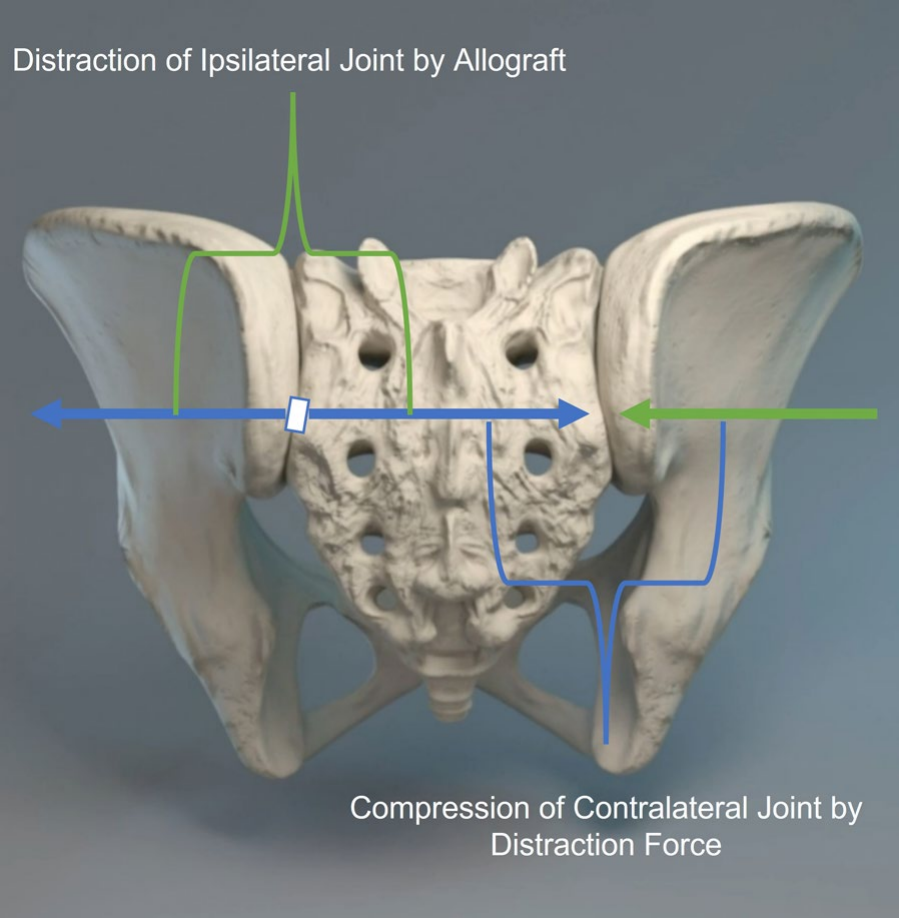
Illustration of pelvis showing contralateral joint stabilization by means on ipsilateral distraction interference

Unilateral fixation using the posterior approach reduced the primary and secondary joints’ mobility in flexion–extension, lateral bending, and axial rotation. Lindsey et al. [10] using the lateral approach reduced the primary and secondary joints’ mobility in only flexion–extension by 46% and 22%, respectively. The previous study reported that the mobility of the pubic symphysis appeared to be the reason that unilateral joint fixation did not reduce motion of the contralateral joint significantly [10]. Our results are somewhat similar, as while reductions in the mobility of the joints were significant in flexion–extension, lateral bending, and axial rotation when pooled together, reductions in mobility of the ipsi and contralateral joint alone in flexion–extension were not significant upon unilateral fixation. This stabilizing effect may be due to the compressive effect which the force generated by the distraction interference of the ipsilateral joint (in the posterior approach) may have on the contralateral joint as described in Fig. 10. This underscores the importance of intra-articular placement of the stabilizing implant, as placement in the recess of the joint at the level of the PSIS as described for the DIANA method may eliminate this stabilizing effect, as the width of the recess makes contralateral stabilization difficult [30]. At the same time, ipsilateral fixation has been shown to be difficult when the implant is placed close to the axis of rotation [31]. While this remains a pitfall of the DIANA technique, the LinQ posterior technique is not placed in the same location as the DIANA method. DIANA places the implant into the joint’s recess, in the region of the interosseous ligament, and enters at the level of the PSIS, which is where the axis of the joint motion is located. In contrast, the allograft implant is placed much lower, i.e., below the PSIS, and is thus farther away from the joint’s axis of rotation.

Bilateral fixation using the posterior approach maintained the reduction in the primary and secondary joints’ mobility in flexion–extension, lateral bending, and axial rotation. Lindsey et al. [10] using the lateral approach reported a 45% and 75% decrease in the primary and secondary joints’ mobility, respectively, from the intact joints’ motion in only flexion–extension, upon bilateral fixation. It is also reported that bilateral fixation maintains the reduced mobility of the primary joint in flexion–extension [10]. Our results are also similar, as bilateral fixation maintained the reduced joint mobility introduced by unilateral fixation in flexion–extension, lateral bending, and axial rotation. However, while we observed significant changes upon bilateral fixation during lateral bending and axial rotation in the secondary joint, we did not observe significant changes in the mobility of the secondary joint in flexion–extension.

The standard pure moment multidirectional bending flexibility model has been consistently and reliably utilized by many investigators for evaluating spinal fusion techniques [32, 33]. However, while it is representative of in vivo motions, such as rise-to-stand, rotation, and bending, it does not accurately represent complexity of typical combined in vivo loading. To mitigate the influence of bone deformation on the range of motion results, optical markers were placed as close as possible to the tested sacroiliac joint [34]. Although the statistical power of our analyses was high in our pooled joint analyses (78–83%), they were low to moderate in our independent primary and secondary joint analyses (42–70%). While low sample sizes are common with many cadaver-based investigations, at the time of this study, specimen availability was severely impacted due to the prevalence of SARS-CoV-2 infection in cadavers [35]. While the amount of motion reduction required to promote fusion is not known, the degrees of motion reduction (1°–2°) obtained are similar between the techniques evaluated in this study. The results were not compared with respect to variations in implant placement. While it would be important to highlight the effectiveness of the implant in its ideal placement, this study aimed to not control the exact location of the implant any more than the guidance provided in the surgical technique. This was to ensure that results were clinically representative of the population utilizing this posterior technique, and thus, an average quantification of performance across these variations is a more realistic evaluation of actual clinical biomechanical performance. It is also important to note as shown in Fig. 5 that both the right and left implants followed identical trajectories. The differences noted are thus due to the asymmetry between the left and right joints, which may not be a significant determinant of biomechanical performance in a bilateral construct, compared to the placement trajectory, and insertion position. As in the instance of left–right asymmetry shown in Fig. 5, the biomechanical performance was identical, at 64%, 65%, and 53% motion reduction in the left joint, and 72%, 68%, and 57% motion reduction in the right joint, during flexion–extension, lateral bending, and axial rotation, respectively. This further underscores the importance of clinicians transitioning from placement within the recess (DIANA) to placement below the recess (LinQ) while taking the gradual learning curve into consideration. Although this study describes the initial stability of this approach, the current model cannot simulate biological changes over time, such as time to fusion, subsidence, or creep. However, investigators of this posterior approach have recently reported efficacies ranging from 66.5 to 76.5% [36, 37]. Investigators have also reported in a multicenter case series, in which the posterior intra-articular technique (LinQ) was utilized as a salvage therapy for the lateral trans-articular technique (iFuse) patients who did not show pain improvement and fusion after 20 ± 8 months postoperation [38]. The investigators reported 77 ± 11% reduction in pain scores upon salvage therapy using the posterior intra-articular technique (LinQ) in all patients after 10 ± 6 months postoperation with evidence of bony bridging.

## Conclusions

The stabilizing effect of the posterior approach in sacroiliac joint arthrodesis had not been previously compared biomechanically to the lateral approach. Our study concludes that during flexion–extension loading, the posterior approach is equivalent to the lateral approach, with additional stabilization during lateral bend, and axial rotation loading, in both unilateral and bilateral SI joint fixation. As in vivo motions are a complex combination of various loads, fixation under multiple moment loads may result in increased efficacy. We also conclude that unilateral joint fixation with the posterior approach is capable of stabilizing both the ipsilateral and contralateral SI joints and that bilateral fixation maintains this stabilizing effect in both joints. Thus, as this approach is minimally invasive, bilateral fixation may be warranted if the fusion of both joints is desired.

## Data Availability

The datasets used and/or analyzed during the current study are available from the corresponding author on reasonable request.
